# Anisakid parasites (Nematoda: Anisakidae) in 3 commercially important gadid fish species from the southern Barents Sea, with emphasis on key infection drivers and spatial distribution within the hosts

**DOI:** 10.1017/S0031182022001305

**Published:** 2022-12

**Authors:** Arne Levsen, Paolo Cipriani, Marialetizia Palomba, Lucilla Giulietti, Julia E. Storesund, Miguel Bao

**Affiliations:** 1 Institute of Marine Research, Bergen, Norway; 2 Sapienza University of Rome, Rome, Italy; 3 Tuscia University, Viterbo, Italy

**Keywords:** *Anisakis*, Arctic, Atlantic cod, Barents Sea, *Contracaecum*, Haddock, Infection drivers, *Pseudoterranova*, Saithe, Spatial distribution

## Abstract

Northeast Arctic cod, saithe and haddock are among the most important fisheries resources in Europe, largely shipped to various continental markets. The present study aimed to map the presence and distribution of larvae of parasitic nematodes in the Anisakidae family which are of socioeconomic and public health concern. Fishes were sourced from commercial catches during winter or spring in the southern Barents Sea. Samples of fish were inspected for nematodes using the UV-press method while anisakid species identification relied on sequencing of the mtDNA *cox2* gene. *Anisakis simplex* (s.s.) was the most prevalent and abundant anisakid recorded, occurring at high infection levels in the viscera and flesh of cod and saithe, while being less abundant in haddock. *Contracaecum osculatum* (s.l.) larvae, not found in the fish flesh, showed moderate-to-high prevalence in saithe, haddock and cod, respectively. Most *Pseudoterranova* spp. larvae occurred at low-to-moderate prevalence, and low abundance, in the viscera (*Pseudoterranova bulbosa*) and flesh (*Pseudoterranova decipiens* (s.s.) and *Pseudoterranova krabbei*) of cod, only 2 *P. decipiens* (s.s.) appeared in the flesh of saithe. Body length was the single most important host-related factor to predict overall abundance of anisakid larvae in the fish species. The spatial distribution of *Anisakis* larvae in the fish flesh showed much higher abundances in the belly flaps than in the dorsal fillet parts. Trimming of the flesh by removing the belly flaps would reduce larval presence in the fillets of these gadid fish species by 86–91%.

## Graphical abstract

**Figure:**
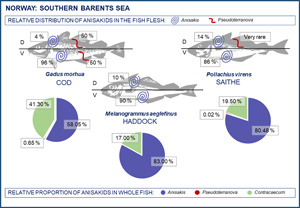

## Introduction

Atlantic cod (*Gadus morhua* L.), saithe (*Pollachius virens* L.) and haddock (*Melanogrammus aeglefinus* L.) are among the commercially most important fish species in European waters. All 3 consist of different stocks, with the Northeast Arctic (NEA) component of each forming the basis for rich fisheries in the Barents Sea region. For example, approximately 375 k tonnes of NEA cod, 190 k tonnes of NEA saithe and 100 k tonnes of NEA haddock landed in Norway in 2021, which together represented more than 10^10^ Norwegian kroner (NOK) in catch value. In fact, the value of catch of these 3 gadids alone was higher than the catch value of all the other commercial fish species combined (9.95^10^ NOK) in 2021 (Directorate of Fisheries, 2022). Fish belonging to these Arctic stocks live as juveniles and/or adults in the oceanic parts of the Barents Sea, while they migrate closer to the coast to spawn in winter or spring (Olsen *et al*., 2010). Members of the NEA cod stock, called ‘skrei’ (from Norse *skreið*, meaning to walk or move) in Norwegian, differ genetically as well as in feeding and spawning behaviour from the local, more stationary coastal cod stock(s) in northern Norway (Skarstein *et al*., 2007). The abundance of ‘skrei’ has increased considerably in recent years, due to a combination of favourable climatic conditions and good management practices (Kjesbu *et al*., 2014).

Although the larvae of anisakid nematodes commonly occur in many commercially important fish species from the NE Atlantic (Mattiucci *et al*., 2017; Levsen *et al*., 2018), information is scarce about the occurrence of this group of parasites in Arctic stocks of Atlantic cod, saithe and haddock. The first to report on nematode/anisakid parasites of Atlantic cod in the Barents Sea was Kahl (1939). More recently, several studies addressed specific aspects of anisakid nematode infections in Atlantic cod from the Barents Sea (Platt, 1975; Aspholm, 1995; Hemmingsen *et al*., 2000; Smith and Hemmingsen, 2003; Sobecka *et al*., 2011; Gay *et al*., 2018; Najda *et al*., 2018; Bao *et al*., 2020), a single study addressing haddock (Pierce *et al*., 2018), while reports on anisakid infections in NEA saithe appear to be absent. Beside the potential of anisakids to negatively affect fish product quality and consumer perception, several species of the genera *Anisakis*, *Contracaecum* and *Pseudoterranova* are zoonotic due to their ability to cause anisakidosis, i.e. direct human infection and/or allergic responses, after consumption of raw or only lightly processed fish (see reviews by Audicana and Kennedy, 2008; Buchmann and Mehrdana, 2016; Mattiucci *et al*., 2017; Bao *et al*., 2019). Anisakids have complex life cycles which generally involve cetaceans (mainly for *Anisakis*) and pinnipeds (mainly for *Contracaecum* and *Pseudoterranova*) as definitive hosts, fish (and squid for *Anisakis*) as paratenic hosts, while planktonic or semi-planktonic crustaceans act as intermediate or paratenic hosts (see review by Mattiucci *et al*., 2017). Most catches of NEA cod, saithe and haddock are shipped to various European export destinations where especially fresh cod has repeatedly received attention due to findings of nematodes in the products (Mercken *et al*., 2020, 2021; Bao *et al*., 2021). Thus, updated information on the presence and site distribution of anisakid larvae could contribute to better assess and manage the risk inflicted by the occurrence of nematodes in these valuable fish resources.

### Aim of the study

The present study aimed to investigate species diversity and spatial distribution of anisakid nematodes in Arctic stocks of Atlantic cod, saithe and haddock, sampled during periods of spawning or feeding migrations in the southern Barents Sea. The study focused on anisakids as potentially zoonotic nematodes whereas the presence of the quality reducing raphidascaridid species *Hysterothylacium aduncum* in basically the same fish host samples was covered in another recent investigation (Bao *et al*., 2021). The present data were analysed with respect to assumably important infection drivers as well as differences and variability of preferred infection sites of actual anisakids in the fish host species.

## Material and methods

**Fig. 1.**
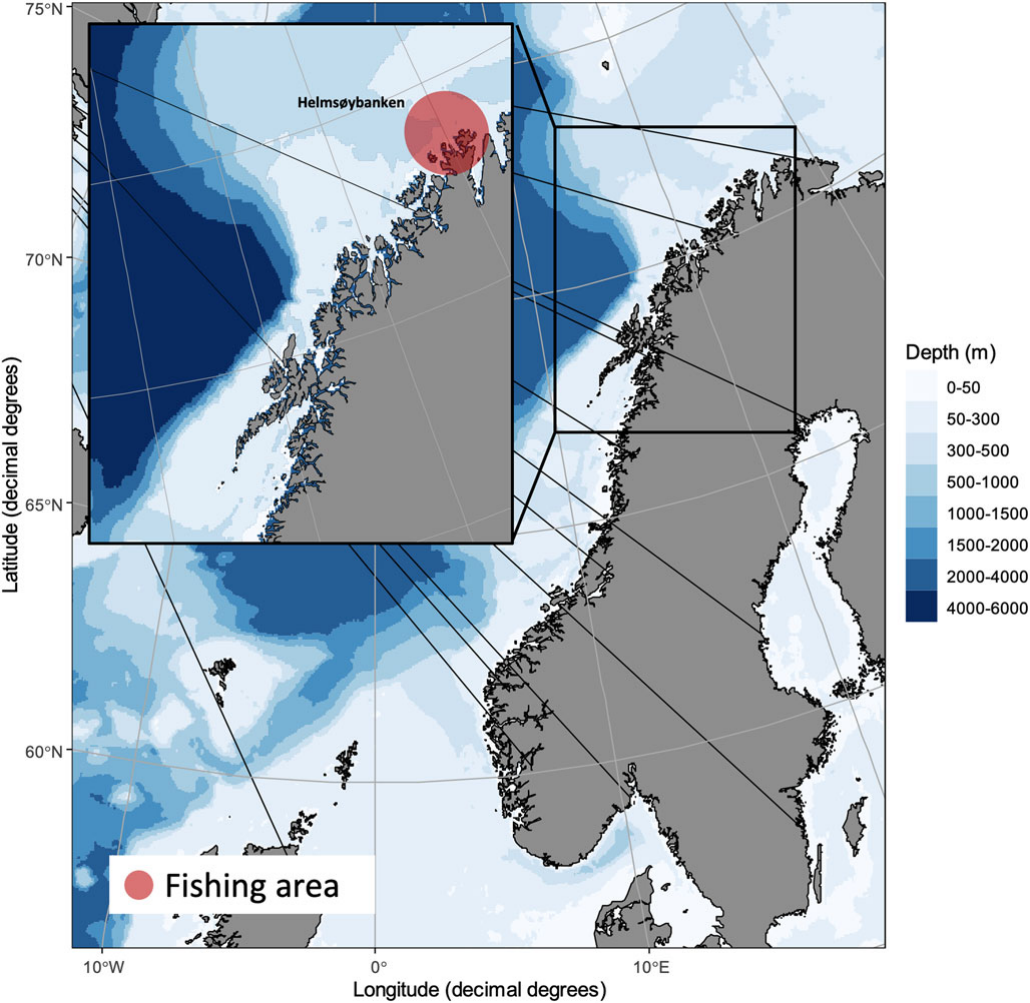
Catching locality of NE Arctic cod, NE Arctic saithe and NE Arctic haddock in the southern Barents Sea.

Gadoids comprising 77 cod, 59 saithe and 60 haddock were caught by local fishermen at ‘Helmsøybanken’ (approximately 71°N 25°E) off West-Finnmark, Norway, during winter or spring 2019 using Danish seine or long-line. The catching localities and catching dates per fish species are shown in Fig. 1. Fishes were frozen shortly after capture and sent to our laboratory for parasitological inspection (see below).

### Fish host biometrics

After thawing, each fish was measured (total body length – TL in mm) and weighed (total body weight – TW in g). Additionally, liver weight (in g) was recorded of each host species and sampling. Subsequently, the hepatosomatic index (HSI) was calculated: 100 × liver weight/total body weight. Details on sample size per catching locality and date, as well as fish biometrics including HSI, are shown in Table 1. Differences in host biometric parameters such as TL, TW and HSI between sampling months of each fish species were assessed by *t*-tests after log-transforming the data.

**Table 1.** Biometric data of NE Arctic cod, NE Arctic saithe and NE Arctic haddock including hepatosomatic index (HSI) and sex ratio, by sampling date

Fish host species	Sampling date		
Atlantic cod	Early February 2019	Mid-March 2019	Late May 2019
Sample size (*N*)	18	32	27
Body length (mm)	1010 ± 58 (880–1080)	775 ± 66 (630–930)	671 ± 70 (500–780)
Body weight (g)	10024 ± 1899 (7240–13700)	4438 ± 1310 (2149–8900)	2419 ± 714 (930–3460)
HSI	10.2 ± 2.4 (6.4–13.4)	6.3 ± 1.5 (2.6–9.2)	3.8 ± 1.7 (1.0–7.7)
Sex ratio ♂/♀	5/13	11/21	13/14
Saithe	Early April 2019	Late May 2019	
Sample size (*N*)	30	29	
Body length (mm)	528 ± 48 (415–610)	605 ± 62 (440–740)	
Body weight (g)	1570 ± 415 (750–2400)	2058 ± 631 (910–3530)	
HSI	9.7 ± 1.3 (7.2–13.1)	8.1 ± 2.2 (3.4–11.8)	
Sex ratio ♂/♀	9 / 21	14 / 15	
Haddock	Early April 2019	Late May 2019	
Sample size (*N*)	30	30	
Body length (mm)	513 ± 49 (420–630)	540 ± 65 (415–640)	
Body weight (g)	1361 ± 441(780–2772)	1375 ± 451 (600–2320)	
HSI	4.8 ± 1.0 (3.4–6.9)	2.3 ± 0.8 (1.2–4.5)	
Sex ratio ♂/♀	8/22	19/11	

Body length, body weight and HSI are given as mean ± s.d. (range).

### Parasitological inspection and anisakid species identification

Fishes were slit open followed by gross visual inspection of the internal organs including mesenteries and peritoneum. Stomach contents were recorded when possible. After filleting, both viscera and each flesh side were placed separately in transparent plastic bags for anisakid inspection by using the UV-press method (ISO 23036-1:2021). In brief, each bag containing flesh sides or viscera was flattened to 2–3 mm thin layers in a hydraulic pressing device for 3–4 s at 10–12 bar, followed by inspection for nematodes under a 366 nm UV-light source equipped with both up- and down-light. In initially frozen fish tissues, any anisakid nematodes present emerge as brightly fluorescent spots, coils or threads when exposed to UV-light. The method further allows to assess the approximate larval infection site in both the fish flesh and viscera (Levsen *et al*., 2018), and even facilitates to grossly distinguish between larvae of *Anisakis*, *Contracaecum* and *Pseudoterranova* species based on differences in brightness, colour and shade of fluorescence (Bao *et al*., 2021). Whenever genus assignment was uncertain, microscopic morphological analysis of anisakid larvae was conducted to verify the genera diagnostic characters, i.e. position of the excretory pore relative to boring tooth and nerve ring, shape and length of ventricle, presence/absence of intestinal caecum, ventricular appendix, terminal mucron or cuticle ornamentation (see Berland, 1961, 1989). After assignment to genus, worms were frozen before further analyses.

Genetic identification was performed on a subsample of 240 randomly selected nematodes comprising specimens of *Anisakis* spp., *Pseudoterranova* spp. and *Contracaecum* spp. obtained from different tissues of the present host species. DNA was extracted using DNeasy Blood & Tissue kits (Qiagen, Hilden, Germany) following the manufacturer's instructions. The mitochondrial cytochrome *c* oxidase subunit II (*cox2*) gene was amplified using the primers 211F (5′-TTTTCTAGTTATATAGATTGRTTTYAT-3′) and 210R (5′-CACCAACTCTTAAAATTATC-3′) (Nadler and Hudspeth, 2000). Polymerase chain reaction (PCR) was carried out by basically following the procedures as outlined by Mattiucci *et al*. (2014). PCR reactions were subjected to the following conditions: 94°C for 5 min, followed by 35 cycles at 94°C for 30 s, 55°C for 60 s, 72°C for 90 s, followed by post-amplification at 72°C for 10 min. Purification and sequencing of PCR products were carried out by Eurofins (Cologne, Germany) using the same primers as those for the amplification. Sequences were assembled using ChromasPro 2.1.5 software (Technelysium Pty Ltd., Tewantin, Australia), edited and trimmed with BioEdit v7.0.5.3, and analysed in GenBank database (BLAST, http://www.ncbi.nlm.nih.gov/BLAST).

### Anisakid infection data analysis

To check for differences in the prevalence of anisakid larvae between sampling months of each host species, Fisher's exact test was applied, while Spearman's rank tests were run to analyse the relationships between host body size (TL) and the abundance of anisakid larvae in overall infections and, whenever relevant, separately for *Anisakis* or *Contracaecum* species larvae in various infection sites. To assess the effect of fish host length (TL), host sex and sampling month on the abundance of anisakid larvae in total, in the viscera and in the flesh, separately for *Anisakis* sp. and *Anisakis*, *Contracaecum* and *Pseudoterranova* species combined (*Pseudoterranova* sp. only for cod), generalized linear model (GLM) procedures were run. In the models, sampling month and host sex were treated as grouping factors while fish body length (TL) was used as a continuous predictor. Fish length and abundance data (*N*) were log-transformed [Log (*N* + 1)] prior to analyses to fit a normal log-link distribution. The goodness of fit of each model was deemed good if the ratio deviance/df was ⩽1. The significance of the contributions of TL, host sex and sampling month on the effect on larval abundance was assessed with likelihood type III tests. Host body weight (TW) was not included as a factor in the models since it may be strongly influenced by short-term changes in gonad and liver weight during periods of spawning or feeding, especially in NEA cod where the combined gonad–liver weight reached up to 32% of the TW in the present samples of February but declined to 1.5% in March. Finally, Kruskal–Wallis analysis of variance followed by 2-tailed comparison of significance (*P* values), or Wilcoxon matched pairs test, was run to compare anisakid abundances between samplings of fish, both per species and comparatively between them. Due to generally high anisakid prevalence in the present fish samples, especially for *Anisakis* and *Contracaecum* species, we decided to use abundance as absolute infection descriptor since, by definition, abundance and intensity are equal at 100% prevalence (see Bush *et al*., 1997). All statistical analyses including data exploration and modelling were performed in STATISTICA v13.4 (TIBCO Software Inc.).

## Results

### Parasite identification

In total, 16 306 anisakid nematode larvae were detected in the present cod samples. According to morphology as well as brightness, colour and shade of fluorescence under UV light, 9526 larvae were morphologically assigned to *Anisakis*, 102 to *Pseudoterranova* and 6678 to *Contracaecum*. In saithe, 9794 anisakid larvae were detected and assigned to *Anisakis* (*n* = 7669), *Pseudoterranova* (*n* = 2) and *Contracaecum* (*n* = 2123). In haddock, 2137 anisakid larvae were recorded and assigned to *Anisakis* (*n* = 1774) and *Contracaecum* (*n* = 363). No *Pseudoterranova* larvae were found in the present haddock samples.

One hundred and forty (140) mtDNA *cox*2 nucleotide gene sequences (571 bp) obtained from larvae morphologically assigned to *Anisakis* matched >99% with a sequence of *Anisakis simplex* (s.s.) from North Sea herring (GenBank accession no. KY595220). Out of the 39 larvae morphologically assigned to *Pseudoterranova*, 13 matched >99% with a sequence of *Pseudoterranova decipiens* sensu stricto (GenBank accession no. MT347695), 6 matched at 98.8% with a sequence of *Pseudoterranova krabbei* (GenBank accession no. HM147279) and 20 matched at 98.5% with a sequence of *Pseudoterranova bulbosa* (GenBank accession no. HM147280). The mtDNA *cox*2 sequences obtained from 60 larvae morphologically assigned to *Contracaecum* matched at 98.6% with a sequence of *Contracaecum osculatum* sp. B (GenBank accession no. MT448514), while only a single larva matched with a sequence of *C. osculatum* sp. A (GenBank accession no. EU477203).

*Anisakis simplex* (s.s.) larvae occurred in the flesh and visceral organs (liver, pyloric caeca, gonads and around the digestive tract) of all fish species examined. *Pseudoterranova decipiens* (s.s.) larvae were found exclusively in the muscle tissue of cod and saithe. *Pseudoterranova krabbei* larvae were recovered only from the muscle tissue of cod whereas *P. bulbosa* larvae were found between the pyloric caeca and in/on the liver of the same fish species. Practically all *C. osculatum* sp. B larvae occurred around the pyloric caeca of the 3 fish species, whereas the single specimen of *C. osculatum* sp. A was recovered from the pyloric caeca of haddock.

Two mtDNA *cox*2 sequences obtained per anisakid species were deposited in GenBank, under accession numbers: OP418109 and OP418110 [*A. simplex* (s.s.)], OP418116 and OP418117 [*P. decipiens* (s.s.)], OP418118 and OP418119 (*P. krabbei*), OP418114 and OP418115 (*P. bulbosa*), OP418112 and OP418113 (*C. osculatum* sp. B), and OP418111 (*C. osculatum* sp. A).

### Fish host characteristics

In NEA cod, mean body size varied significantly between sampling months, i.e. both total length and weight were significantly different in all 3 sampling months (*t*-tests, *P* < 0.001), with the fish sampled in February being by far biggest, barely overlapping in size with the samples of March and May. There was a strongly significant linear length–weight relationship in cod for all 3 sampling months combined (*r* = 0.96, *P* < 0.0001). Additionally, the HSI varied significantly between sampling months (*t*-tests, *P* < 0.001), with cod sampled in February reaching by far the highest value (Table 1).

As for cod, there was a strong linear relationship between total body length and weight of saithe for all samplings combined (*r* = 0.91, *P* < 0.0001).

NEA haddock showed nearly equal body size distribution across the 2 samplings, including TL and TW (Table 1). The HSI was the only fish size-related parameter that differed significantly between the samples of April and May (*t*-test, *P* < 0.001). As for cod and saithe, there was a strongly significant linear length–weight relationship in the 2 haddock samples (*r* = 0.91, *P* < 0.0001). Comparatively across all host species and samplings, haddock had a lower HSI than cod and saithe (*P* < 0.001).

### Anisakid infection characteristics

#### NEA cod

**Fig. 2.**
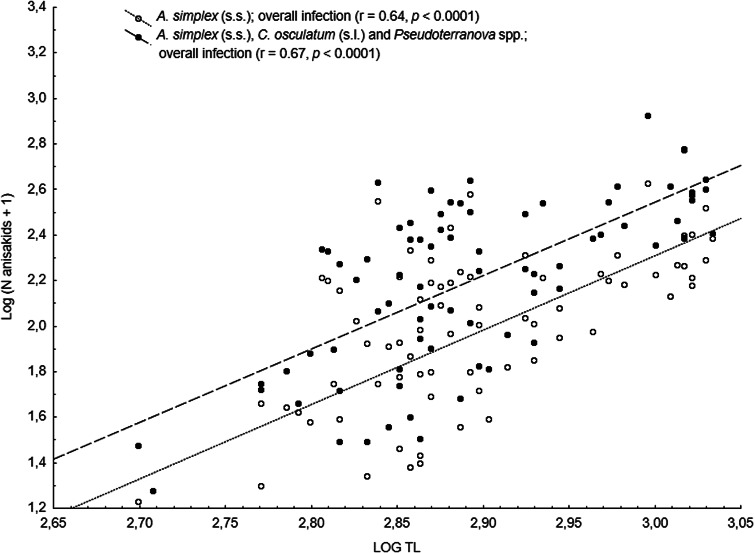
Overall abundance of *A. simplex* (s.s.) and *A. simplex* (s.s.), *C. osculatum* (s.l.) and *Pseudoterranova* spp. larvae combined, in NE Arctic cod as a function of fish total length (TL) (data were log-transformed).

**Fig. 3.**
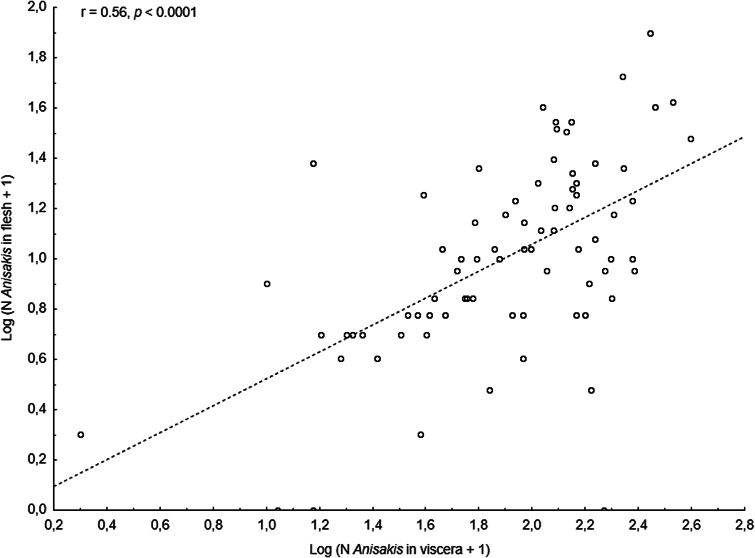
Relationship between abundance of *A. simplex* (s.s.) larvae in the viscera and in the flesh of NE Arctic cod (data were log-transformed).

The main anisakid infection descriptors per infection site and sampling month are shown in Table 2. The prevalence of *A. simplex* (s.s.) and *C. osculatum* (s.l.) larvae was total (100%) in the viscera of cod in all samples and even reached >90% for *A. simplex* (s.s.) in the fish flesh. The overall prevalence of *Pseudoterranova* spp. varied significantly between the cod samples of February and May while the prevalence of these larvae in the cod flesh [*P. decipiens* (s.s.) and *P. krabbei*], being uninfected in February, did not differ significantly between the samples of March and May. In pooled samples covering all 3 sampling months, there was a significantly positive correlation between cod body length (TL) and overall *A. simplex* (s.s.) abundance (*N* larvae in both viscera and flesh; *r* = 0.64, *P* < 0.001). A similar correlation was found between TL and overall abundance of all 3 larval anisakids combined [*A. simplex* (s.s.), *C. osculatum* (s.l.) and *Pseudoterranova* spp.; *r* = 0.67, *P* < 0.001] (Fig. 2). However, there was no clear relationship between cod TL and abundance of *A. simplex* (s.s.) larvae in the fish flesh in either month, except of May where these variables were only weakly correlated (*r* = 0.44, *P* < 0.029). Another significantly positive relationship was found between the abundance of *A. simplex* (s.s.) larvae in the viscera and flesh (*r* = 0.56, *P* < 0.001) (Fig. 3).

**Table 2.** Prevalence, mean abundance and abundance range of anisakid nematode species from NE Arctic cod (A), NE Arctic saithe (B) and NE Arctic haddock (C), per infection site and sampling month.

A. NE Arctic cod		*Anisakis simplex* (s.s.)				*Contracaecum osculatum* (s.l.)				*Pseudoterranova* spp.			
Infection parameters	Site	February	March	May	All months	February	March	May	All months	February	March	May	All months
Prevalence (%)	Flesh Viscera Total	94.4 100 100	100 100 100	92.6 100 100	96.1 100 100	0 100 100	0 100 100	0 100 100	0 100 100	0 16.7 16.7	25.0 15.6 34.4	37.0 22.2 55.6	23.4 18.2 37.7
Abundance (mean ± s.d.)	Flesh Viscera Total	12 ± 10 190 ± 75 201 ± 82	13 ± 10 83 ± 48 96 ± 53	15 ± 18 90 ± 87 105 ± 102	13.5 ± 13 110 ± 82 124 ± 90	0 179 ± 114 179 ± 114	0 67 ± 55 67 ± 55	0 52 ± 43 52 ± 43	0 88 ± 86 88 ± 86	0 0.4 ± 0.9 0.4 ± 0.9	0.9 ± 2.3 0.5 ± 1.2 1.4 ± 2.8	1.6 ± 3.2 0.3 ± 0.7 1.9 ± 3.3	0.9 ± 2.5 0.4 ± 1.0 1.3 ± 2.7
Abundance range	Flesh Viscera Total	0–39 83–395 88–424	2–39 14–196 21–205	0–78 1–338 2–379	0–78 1–395 2–424	0 13–421 13–421	0 5–190 5–190	0 2–165 2–165	0 2–421 2–421	0 0–3 0–3	0–10 0–4 0–11	0–14 0–3 0–14	0–14 0–4 0–14

B. NE Arctic saithe		*Anisakis simplex* (s.s.)			*Contracaecum osculatum* (s.l.)		
Infection parameters	Site	April	May	All months	April	May	All months
Prevalence (%)	Flesh Viscera Total	100 100 100	100 100 100	100 100 100	0 100 100	0 100 100	0 100 100
Abundance (mean ± s.d.)	Flesh Viscera Total	15.5 ± 12 91 ± 51 106 ± 60	20 ± 15.5 135 ± 82 155 ± 90	18 ± 14 112 ± 71 130 ± 79.5	0 38 ± 21 38 ± 21	0 34 ± 20.5 34 ± 20.5	0 36 ± 21 36 ± 21
Abundance range	Flesh Viscera Total	2–61 22–199 25–260	1–72 22–354 24–393	1–72 22–354 24–393	0 9–87 9–87	0 5–109 5–109	0 5–109 5–109

C. NE Arctic haddock		*Anisakis simplex* (s.s.)			*Contracaecum osculatum* (s.l.)		
Infection parameters	Site	April	May	All months	April	May	All months
Prevalence (%)	Flesh Viscera Total	76.7 100 100	66.7 96.7 96.7	71.7 98.3 98.3	0 80.0 80.0	0 86.7 86.7	0 83.3 83.3
Abundance (mean ± s.d.)	Flesh Viscera Total	2.3 ± 2.5 24.5 ± 16 27 ± 17	1.8 ± 2.6 31 ± 24 33 ± 25	2.0 ± 2.5 27.5 ± 20.5 29.5 ± 21.5	0 7.1 ± 7.5 7.1 ± 7.5	0 5.0 ± 4.8 5.0 ± 4.8	0 6.1 ± 6.3 6.1 ± 6.3
Abundance range	Flesh Viscera Total	0–11 3–76 4–83	0–12 0–90 0–96	0–12 0–90 0–96	0 0–30 0–30	0 0–23 0–23	0 0–30 0–30

The results of the GLM modelling of larval anisakid abundance are shown in Table 3. The modelling revealed that TL had by far the strongest effect on *A. simplex* (s.s.) abundance in the models for overall abundance (*P* < 0.001) and abundance in the viscera of cod (*P* < 0.001) but was very weak for larval abundance in the flesh (*P* < 0.042). Sampling month was shown to be only a weak predictor of *A. simplex* (s.s.) abundance in the models for overall infection (*P* = 0.034) and the visceral organs (*P* = 0.013). A similar pattern was seen in the model for overall abundance of *Anisakis*, *Contracaecum* and *Pseudoterranova* species combined, with TL as a highly significant explanatory factor (*P* < 0.001) while sampling month had only a very weak effect (*P* = 0.048). Host sex was not significant as a predictor of larval anisakid abundance in either model.

**Table 3.** Results of GLM modelling of larval anisakid abundance in various infection sites per fish host species.

Anisakid species	*A. simplex* (s.s.)									*A. simplex* (s.s.), *C. osculatum* (s.l.), *Pseudoterranova* spp.		
GLM model	Overall abundance (viscera + flesh)			Abundance in viscera			Abundance in flesh			Overall abundance (viscera + flesh)		
Atlantic cod	d.f.	*χ*^2^	*P*	d.f.	*χ*^2^	*P*	d.f.	*χ*^2^	*P*	d.f.	*χ*^2^	*P*
Total length (TL)	1	26.9	**<0.001**	1	32.4	**<0.001**	1	4.1	**0.042**	1	25.7	**<0.001**
Sampling month	2	6.7	**0.034**	2	8.6	**0.013**	2	4.2	0.123	2	6.1	**0.048**
Sex	1	0.08	0.777	1	0.009	0.925	1	1.3	0.250	1	0.2	0.648
Saithe	*A. simplex* (s.s.)									*A. simplex* (s.s.), *C. osculatum* (s.l.)		
Total length (TL)	1	37.9	**<0.001**	1	40.5	**<0.001**	1	9.12	**0.003**	1	40.9	**<0.001**
Sampling month	1	3.31	0.069	1	3.75	0.053	1	0.51	0.477	1	4.96	**0.026**
Sex	1	0.11	0.747	1	0.12	0.734	1	0.08	0.773	1	0.26	0.608
Haddock	*A. simplex* (s.s.)									*A. simplex* (s.s.), *C. osculatum* (s.l.)		
Total length (TL)	1	7.1	**0.008**	1	7.5	**0.006**	1	0.13	0.717	1	6.8	**0.009**
Sampling month	1	0.65	0.420	1	0.52	0.470	1	0.26	0.608	1	1.1	0.304
Sex	1	0.92	0.337	1	1.3	0.253	1	0.71	0.401	1	0.64	0.425

d.f., degree of freedom; *P*, significance level.

Significant effects are highlighted.

The considerably larger body size of cod sampled in February was also reflected by differences between the sampling months with respect to the abundance of anisakid larvae in various infection sites of cod. Thus, overall abundance and abundance in the viscera of *A. simplex* (s.s.) and *C. osculatum* (s.l.) larvae in cod sampled in February, both separately and combined, was significantly higher than in the samples obtained in March and May (*P* < 0.001 in all cases). Likewise, the overall abundance of the present anisakids combined [*A. simplex* (s.s.), *C. osculatum* (s.l.) and *Pseudoterranova* spp.] was significantly higher in February than in the samples of March and May (*P* < 0.001). However, neither the abundance of *A. simplex* (s.s.) larvae in the fish flesh nor the abundance of *Pseudoterranova* spp. larvae in the viscera differed significantly between the cod samples of the 3 sampling months.

#### NEA saithe

**Fig. 4.**
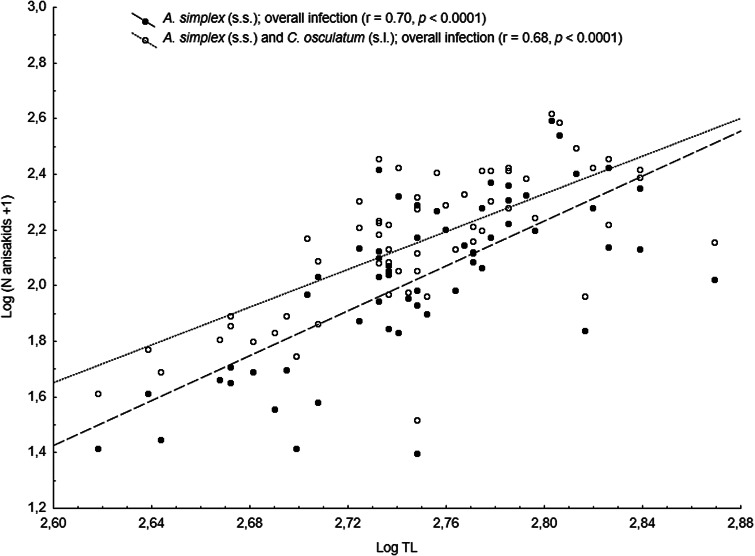
Overall abundance of *A. simplex* (s.s.) and *A. simplex* (s.s.) and *C. osculatum* (s.l.) larvae combined in NE Arctic saithe as a function of fish total length (TL) (data were log-transformed).

**Fig. 5.**
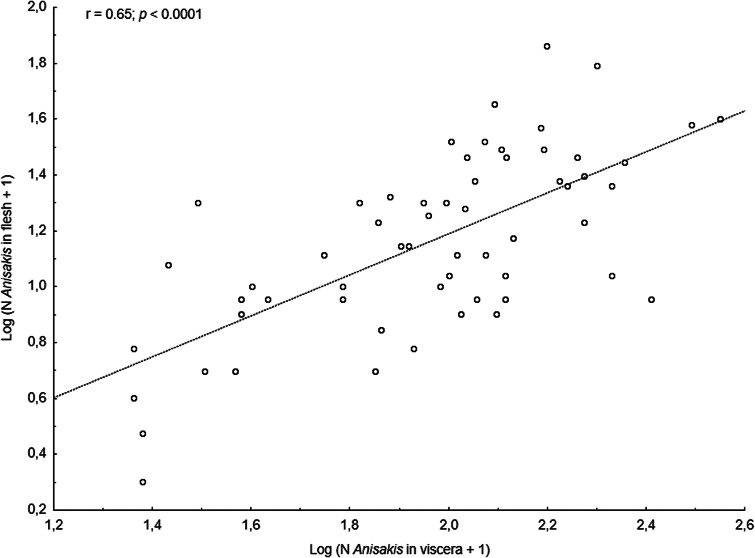
Relationship between abundance of *A. simplex* (s.s.) larvae in the viscera and in the flesh of NE Arctic saithe (data were log-transformed).

*Anisakis simplex* (s.s.) and *C. osculatum* sp. B appeared to be the dominating anisakids in saithe, both species reaching total prevalence (100%) in all samplings and infection sites (Table 2). Two individual *P. decipiens* (s.s.) larvae were recorded in the dorsal musculature of a single saithe caught in May 2019. There were comparatively strongly positively linear correlations between saithe body length (TL) and overall larval abundances, both separately for *A. simplex* (s.s.) (*r* = 0.70, *P* < 0.001) and *A. simplex* (s.s.) and *C. osculatum* (s.l.) larvae combined (*r* = 0.68, *P* < 0.001) (Fig. 4). A similarly comparatively strong relationship was found between *A. simplex* (s.s.) larval abundance in the viscera and in the flesh of saithe (*r* = 0.65, *P* < 0.001) (Fig. 5).

GLM modelling showed that host size (TL) was by far the strongest predictor of anisakid larval abundance in all models tested (*P* < 0.003) (Table 3). Sampling month had only a weak effect on overall abundance of *A. simplex* (s.s.) and *C. osculatum* (s.l.) larvae combined (*P* = 0.026). As for the NEA cod models, host sex was not a significant predictor of larval anisakid abundance in either model run for saithe.

#### NEA haddock

Neither prevalence nor abundance of *A. simplex* (s.s.) and *C. osculatum* (s.l.) larvae, separately or combined for any infection site, differed significantly between the 2 samplings of haddock (Table 2). There were only comparatively weakly positive relationships between haddock body length (TL) and overall larval abundance, both separately for *A. simplex* (s.s.) (*r* = 0.37, *P* = 0.004) and *A. simplex* (s.s.) and *C. osculatum* (s.l.) larvae combined (*r* = 0.36, *P* = 0.005). Additionally, and somewhat contrasting cod and saithe, there was only a weakly positive correlation between the abundance of *A. simplex* (s.s.) larvae in the viscera and in the flesh of the fish (*r* = 0.32, *P* = 0.013). GLM modelling revealed that fish length had a much weaker effect on the abundance of the 2 larval anisakid species in haddock compared to cod and saithe (Table 3), which was even non-significant with respect to the abundance of *A. simplex* (s.s.) larvae in the fish flesh. Neither sampling month nor fish host sex were significant predictors of larval anisakid abundance in the present haddock samples (Table 3).

### Infection parameters across fish hosts

**Fig. 6.**
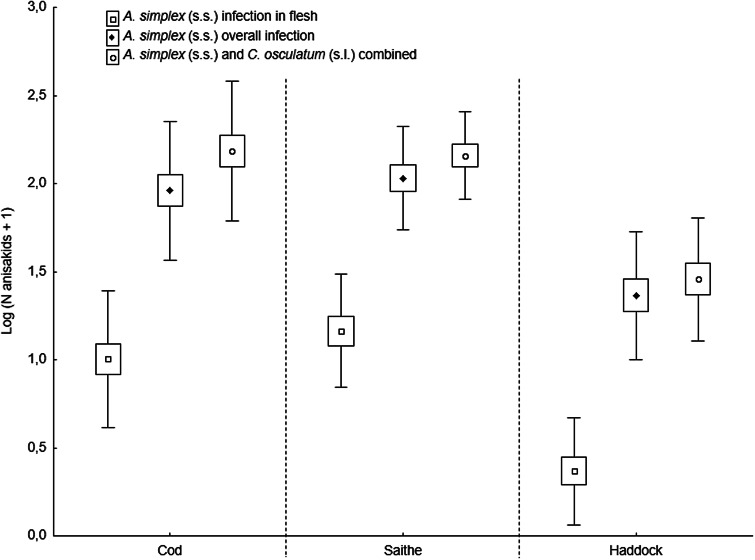
Abundance of *A. simplex* (s.s.) in total and in the flesh, and total abundance of *A. simplex* (s.s.) and *C. osculatum* (s.l.) combined, in 3 gadid fish species from the southern Barents Sea (counts were log-transformed). Abundances are given as mean ± CI ± s.d.

The present fish host species showed generally high prevalence of *Anisakis* and *Contracaecum* species larvae in various body parts. However, the prevalence of *A. simplex* (s.s.) larvae was significantly lower in the flesh of haddock than in cod and saithe (Fisher's exact test; *P* < 0.001), which was also the case for the overall prevalence of *C. osculatum* (s.l.) larvae (Fisher's exact test; *P* < 0.001). Similarly, haddock showed significantly lower abundances of *A. simplex* (s.s.) and *A. simplex* (s.s.) and *C. osculatum* (s.l.) combined than cod and saithe (*P* < 0.001) (Table 2, Fig. 6). For *C. osculatum* (s.l.) alone, overall abundance differed widely between the fish host species (*P* < 0.001), being by far highest in cod (Table 2).

### Spatial distribution of anisakids

**Fig. 7.**
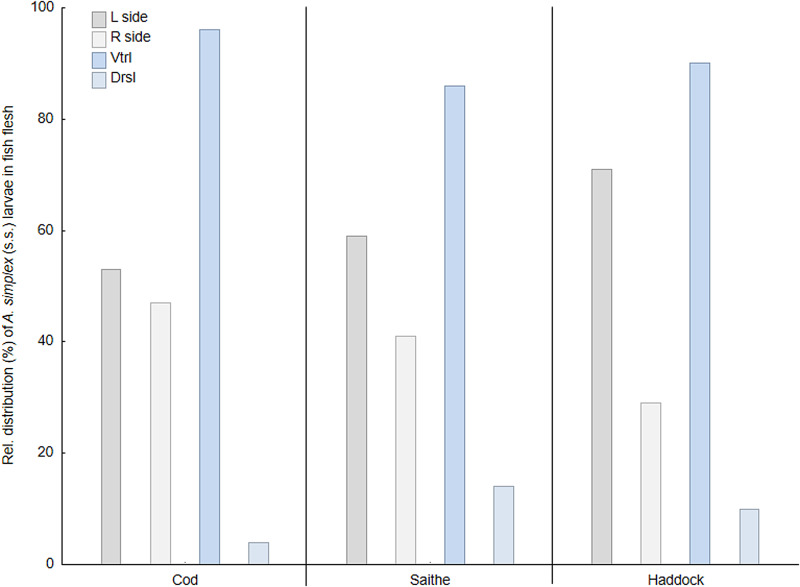
Relative distribution (%) of *A. simplex* (s.s.) larvae in the flesh of 3 gadid fish species from the southern Barents Sea (L side, left flesh side; R side, right flesh side; Vtrl, ventral fillet part; Drsl, dorsal fillet part).

Table 4 shows the relative distribution of anisakid larvae in various parts of the flesh and viscera in the present gadid fish species. In general, *A. simplex* (s.s.) larvae appeared to be more abundant in the left flesh side than in the right side. This trend was especially pronounced in haddock and saithe (Wilcoxon matched pairs test; *P* < 0.001) while non-significant in cod when considering all 3 samplings combined (*P* = 0.09). However, larval abundance was slightly higher in the left flesh side of the March sample of cod (*P* = 0.01) (Table 4). A clear distribution pattern was seen when comparing larval abundance in the ventral and dorsal parts of each flesh side, with the ventral parts largely comprising the belly flaps in each of the present fish species. Thus, more than 85% of all *A. simplex* (s.s) larvae recorded in the fish flesh resided in the belly flaps, even reaching ⩾97% in 2 samples of cod (Table 4). Figure 7 shows the relative distribution (%) of *A. simplex* (s.s.) larvae per flesh side (left/right) and fillet part (ventral/dorsal) in the present fish species for all samplings combined.

**Table 4.** Relative distribution of larval anisakid species in the flesh and viscera of 3 gadids from NE Arctic waters

	Rel. distribution in fish flesh (%)			Rel. distribution in viscera (%)		
Atlantic cod	L side:R side	Vtrl:Drsl	Flesh:viscera	Liver	Pyloric area	Rest viscera
*A. simplex* (s.s.)						
February	47:53	91:9	6:94	22	39	39
March	57:43	97:3	13:87	42	29	29
May	51:49	98:2	15:85	46	17	37
All months	**53**:**47**	**96**:**4**	**11**:**89**	**35**	**30**	**35**
*C. osculatum* (s.l.)						
February	np	np	np	np	94	6
March	np	np	np	np	98	2
May	np	np	np	np	99	1
All months	np	np	np	np	**96.5**	**3.5**
*Pseudoterranova* spp.						
February	np	np	np	43	57	0
March	63:37	57:43	67:33	40	60	0
May	45:55	45:55	84:16	88	12	0
All months	**53**:**47**	**50**:**50**	**71**:**29**	**53**	**47**	**0**
Saithe	L side:R side	Vtrl:Drsl	Flesh:viscera	Liver	Pyloric area	Rest viscera
*A. simplex* (s.s.)						
April	60:40	86:14	15:85	65	15	20
May	58:42	86:14	13:87	69	13	18
All months	**59**:**41**	**86**:**14**	**14**:**86**	**67**	**14**	**19**
*C. osculatum* (s.l.)						
April	np	np	np	np	99	1
May	np	np	np	np	98	2
All months	np	np	np	np	**98.5**	**1.5**
Haddock	L side:R side	Vtrl:Drsl	Flesh:viscera	Liver	Pyloric area	Rest viscera
*A. simplex* (s.s.)						
April	71:29	90:10	8:92	63	27	10
May	72:28	91:9	6:94	70	19	11
All months	**71**:**29**	**90**:**10**	**7**:**93**	**67**	**23**	**10**
*C. osculatum* (s.l.)						
April	np	np	np	np	99	1
May	np	np	np	np	99	1
All months	np	np	np	np	**99**	**1**

L, left; R, right; Vtrl, ventral flesh part; Drsl, dorsal flesh part; np, not present.

Average ratios per parasite species for all sampling months under each fish host species are highlighted in bold.

**Fig. 8.**
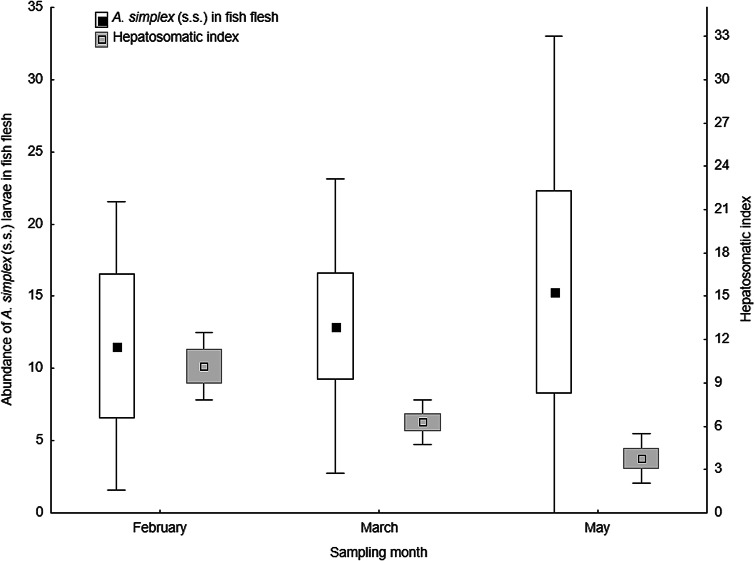
Abundance of *A. simplex* (s.s.) larvae in the flesh and the hepatosomatic index (HSI) of NE Arctic cod per sampling month. Abundance and HSI are given as mean ± CI ± SD.

The relative proportion of *A. simplex* (s.s.) larvae that migrated into the flesh of the fish hosts varied between 6% in single samples of cod and haddock, and 15% in single samples of cod and saithe, being generally lowest in haddock. In cod, the smaller fish of the March and May samples tended to carry relatively more larvae in the flesh than the fish caught in February (Table 4). This trend, also seen for the abundance data (Table 2), coincided with a significant decline in HSI in the cod samples from February to May (Fig. 8).

The distribution of *Pseudoterranova* spp. larvae in the flesh of cod [*P. decipiens* (s.s.) and/or *P. krabbei*] differed from *A. simplex* (s.s.) in several respects. Thus, *Pseudoterranova* spp. were altogether absent from the fish flesh in the strict winter sample of cod (February) while, after their appearance in March, generally half of the larvae were recorded in the dorsal parts of the fish flesh which largely comprise the fillet loins (Table 4). Moreover, by far most *Pseudoterranova* spp. were apparently residing in the fish flesh, which is the opposite of the pattern seen for the present *A. simplex* (s.s.) larvae. No *C. osculatum* (s.l.) larvae were recorded in the flesh of any of the gadid fish host species presently examined.

**Fig. 9.**
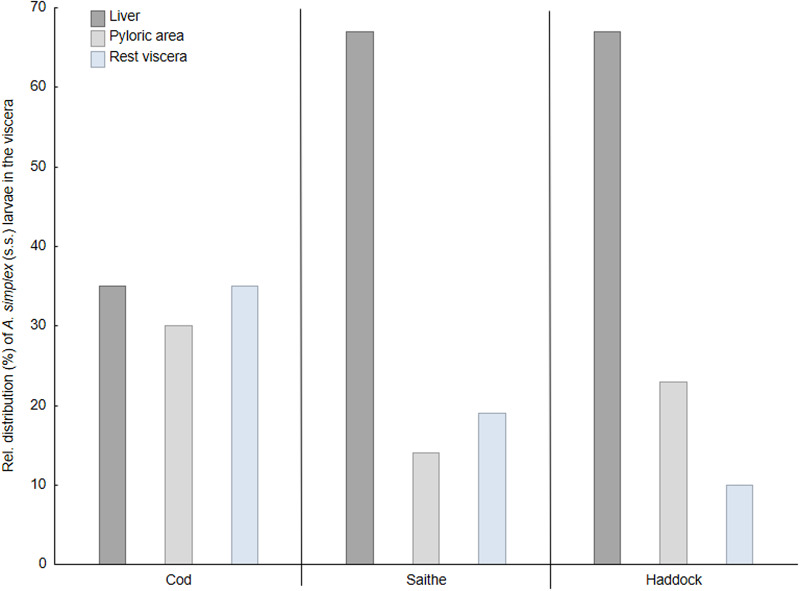
Relative distribution (%) of *A. simplex* (s.s.) larvae in the visceral organs of 3 gadid fish species from the southern Barents Sea.

The liver was the single most important infection site of *A. simplex* (s.s.) in saithe and haddock, carrying two-third of all larvae recorded in the visceral cavity organs, while this proportion was roughly one-third in cod (Fig. 9). Due to generally low abundance of *Pseudoterranova* spp. larvae in the viscera of cod, any reliable assessment of the relative distribution in the visceral cavity organs could not be made. However, there appeared to be a trend towards higher relative *Pseudoterranova* spp. (likely *P. bulbosa*) burden in the liver with increasing season, i.e. from winter to late spring (Table 4). *Contracaecum osculatum* (s.l.) larvae were almost exclusively recorded within or around the pyloric caeca of each of the 3 present fish host species.

## Discussion

### Anisakid species diversity

Genetic identification of subsamples of anisakid larvae revealed the presence of 6 species in the examined gadid hosts, i.e. *A. simplex* (s.s.), *C. osculatum* A, *C. osculatum* B, *P. decipiens* (s.s.), *P. krabbei* and *P. bulbosa*. While the larvae of *A. simplex* (s.s) and *C. osculatum* (s.l.) occurred in all 3 fish hosts, *Pseudoterranova* spp. larvae were mainly infecting cod, with only 2 specimens recorded in saithe, all identified as *P. decipiens* (s.s.). The larval stage of these nematode species showed different site preferences in the hosts, apparently independent of fish species.

*Anisakis simplex* (s.s.) appears to be the only member of the *A. simplex* species complex present in fishes from arctic or subarctic areas of the NE Atlantic including the Norwegian and Barents Seas (Mattiucci *et al*., 2018). It was recently identified in muscle samples of fresh NEA cod intended for stockfish production (Bao *et al*., 2020). The closely related sibling *Anisakis pegreffii* was recently recorded in the fillets of 3 individual Atlantic cod from the northern North Sea (Gay *et al*., 2018). Although rarely recorded in fish hosts outside its distribution range, the species has so far not been detected at the adult stage in its definitive hosts in NE Atlantic waters north of the Bay of Biscay (Mattiucci *et al*., 2018; Cipriani *et al*., 2022). Hence, for the time being, it seems unlikely that *A. pegreffii* occurs in fish species endemic to the Barents Sea.

Two members of the *C. osculatum* species complex, i.e. *C. osculatum* sp. A and *C. osculatum* sp. B, were identified in subsamples taken from each fish species, however, only a single specimen of the former species was found in 1 haddock, in sympatry with *C. osculatum* sp. B. Due to the comparatively small number of *Contracaecum* species larvae that were molecularly identified, one cannot rule out that *C. osculatum* sp. A also occurs in cod or saithe, or that other members of this species complex may parasitize the present fish species, as well. Levsen *et al*. (2016) found comparatively high infection levels of *C. osculatum* sp. B in capelin (*Mallotus villosus* Müller) from basically the same area of the Barents Sea. Capelin comprises one of the most important prey items of larger specimens of cod, saithe and haddock (Olsen *et al*., 2010) during winter and spring, which may at least partially explain the presence and high abundances of *C. osculatum* sp. B in the present host species. However, this *C. osculatum* sibling species, together with *C. osculatum* sp. A and *C. osculatum* (s.s.), has also been identified from Greenland halibut (*Reinhardtius hippoglossoides* Walbaum), caught in more centrally located areas of the Barents Sea (Karpiej *et al*., 2013; Najda *et al*., 2018).

The 3 *Pseudoterranova* species presently identified from cod have all been recorded in fishes from the Barents Sea before. Hence, *P. bulbosa* was found at varying prevalence and abundances in the viscera of Greenland halibut, long rough dab (*Hippoglossoides platessoides* Fabricius), roughhead grenadier (*Macrourus berglax* Lacepède) and Atlantic cod (Karpiej *et al*., 2013; Najda *et al*., 2018) while the presence of *P. decipiens* (s.s.) and *P. krabbei* is documented for Atlantic cod, *P. decipiens* (s.s.) in tusk (*Brosme brosme* Ascanius) and European smelt (*Osmerus eperlanus* L.) (Mattiucci and Nascetti, 2008). Additionally, we molecularly identified *P. decipiens* (s.s.) in dried fillets of NEA cod taken off the Lofoten archipelago in northern Norway (Bao *et al*., 2020).

### Anisakid infection characteristics

The present abundance levels are by far the highest ever recorded in NEA cod. Another recent study of anisakids in Barents Sea cod (Gay *et al*., 2018) which also relied on the UV-press method reported 100% prevalence but much lower overall abundances of *A. simplex* (s.s.) and *C. osculatum* (s.l.) larvae (i.e. 36.88 and 1.96, respectively) in fish that corresponded in size to the present cod samples of May. Other studies reporting on anisakid infections in Barents Sea cod were based either on small sample size and unspecified fish size (Najda *et al*., 2018) or investigated rather small fish not exceeding 500 mm in TL (Sobecka *et al*., 2011). Parasite detection in both studies was based on plain visual inspection, i.e. not applying the UV-press method, which clearly impedes comparison with the present findings. However, Najda *et al*. (2018) reported total prevalence of *A. simplex* (s.s.) and *C. osculatum* (s.l.), while half of the cod were infected with *P. bulbosa*. The overall mean abundance of *Anisakis* was high (i.e. 126.7), reaching similar levels as the present study, while mean abundance of *C. osculatum* (s.l.) appeared to be considerably lower (i.e. 13.0).

High overall prevalence of *A. simplex* (s.s.) was also recorded in saithe and haddock, and for *C. osculatum* (s.l.) in saithe. The larvae of the latter were consistently less prevalent in haddock. On the other hand, saithe showed highest infection levels with *A. simplex* (s.s.) in the flesh of the gadid species presently examined. Both overall abundance and abundance in the flesh of *A. simplex* (s.s.) were particularly high in saithe in May, tending to be even higher than in cod caught at the same time. Strømnes and Andersen (1998) investigated the occurrence of the *A. simplex* (s.s.) in 3 marine fish species including saithe from the Island of Vega off mid-Norway. Although occurring at very high prevalence (97.2%), overall larval abundance was low, with only a few *Anisakis* larvae detected in the fish muscle (checked by plain visual inspection only). Since no other anisakid species were recorded by them, the present findings are the first to report infection data for *C. osculatum* sp. B and *P. decipiens* (s.s.), and, thus, represent new records for saithe.

A recent study of haddock from the Barents Sea (Pierce *et al*., 2018), also based on the UV-press method for parasite inspection, found almost identical prevalence and abundance levels of *A. simplex* (s.s.), both in total and in the flesh, compared with the present results. However, both prevalence and abundance of *C. osculatum* (s.l.) larvae were considerably lower in the former study although they (Pierce *et al*., 2018) found light infection with *Pseudoterranova* spp. larvae in the viscera of the fish, which were absent in haddock of the present study. Since the fish of the former study were slightly bigger, the lower infection level of *C. osculatum* (s.l.) may relate to differences in exposure over time to infective larvae in more coastal localities such as Helmsøybanken, compared to the oceanic areas of the western or central Barents Sea which represented the sampling localities of NEA cod and haddock examined by Gay *et al*. (2018) and Pierce *et al*. (2018), respectively.

The presently observed strong relationship between overall anisakid abundance and fish body length, along with the GLM analyses, clearly showed that body size (TL) represents the single most important host-related variable to predict overall anisakid abundances in the 3 fish host species. Although considerably weaker for the model of TL and *Anisakis* larvae in the fish flesh, and even absent for haddock, the findings still imply that these gadids seem to accumulate *A. simplex* (s.s.) and *C. osculatum* (s.l.) larvae over time. Body size as an important driver of *A. simplex* (s.l.) prevalence and abundance has been reported for many fish species and areas including cod and haddock from the Barents Sea and adjacent waters (Hemmingsen *et al*., 2000; Münster *et al*., 2015; Gay *et al*., 2018; Pierce *et al*., 2018). In general, the apparent accumulation of anisakids in fish over time can be largely explained by an increased piscivorous feeding behaviour with increasing fish size, combined with the comparatively long lifespan of these parasites. For example, all present gadid species feed heavily on capelin in late winter and spring when moving closer to the coast of Finnmark to feed and spawn (Olsen *et al*., 2010). Capelin has been shown to be an important paratenic host for *A. simplex* (s.s.) and *C. osculatum* sp. B in the area, transferring the larvae upward the food web (Levsen *et al*., 2016; Bao *et al*., 2021). Beside the seasonal feeding on capelin, the diet of NEA haddock consists to a larger extent of benthic organisms compared to cod and saithe which both rely on fish prey such as herring and various smaller gadoids, or even feed cannibalistically (Olsen *et al*., 2010). The haddock examined by Pierce *et al*. (2018) were sampled in autumn in oceanic parts of the Barents Sea where they prey on benthic organisms or small fish other than capelin which, however, may not be equally important as paratenic hosts for *C. osculatum* (s.l.). The differences in feeding behaviour between NEA cod and saithe on the one side, and haddock on the other side, may be an important explanatory factor for the differences in anisakid infection levels, especially with respect to *C. osculatum* (s.l.), observed in the 3 fish species.

Other factors that possibly affect infection level and variability of anisakids in fish are linked to the concurrent occurrence of infected intermediate, paratenic and definitive hosts in given areas, the lifespan of actual larvae as well as the ability of individual fish to cope with the infections. Since various cetaceans commonly occur in offshore waters of the Barents Sea, some all year round while others reach these northern latitudes during warmer seasons (Bogstad *et al*., 2015; Mattiucci *et al*., 2018; Mishin, 2021), large volumes of *Anisakis* species larvae persist in the ecosystem, hence, maintaining infections in planktivorous and piscivorous fish. Similarly, harp seal (*Pagophilus groenlandicus*) appears to be one of the most important definitive hosts for *C. osculatum* sp. B in the Barents Sea (Nascetti *et al*., 1993), thus contributing to maintain the life cycle of the parasite in the area. Since harp seal is the most numerous seal species in these waters and seems to remain in the area throughout its lifespan (ICES, 2011), a more or less evenly output of *C. osculatum* sp. B larvae into the ecosystem may contribute to maintain comparatively high infection levels in various key paratenic fish hosts including capelin and the 3 actual gadid species.

*Pseudoterranova decipiens* (s.s.) and *P. krabbei*, on the other hand, both seem to utilize common seal (*Phoca vitulina*) and grey seal (*Halichoerus gryptus*) as definitive hosts, while bearded seal (*Erignatus barbatus*) acts as a definitive host for *P. bulbosa* (Mattiucci *et al*., 2017). All 3 seal species prefer more coastal habitats compared to harp seal which, except during periods of breeding and nursing, lives in more open waters of its distribution range (Nordøy *et al*., 2008; ICES, 2011). Thus, the presently observed infection pattern of *Pseudoterranova* spp. in NEA cod may indicate that they acquire increasing numbers of larvae during migration and spawning along the coast of northern Norway in late winter and spring. Alternatively, the apparently higher infection level of *Pseudoterranova* spp. larvae in March and May may suggest that a certain fraction of the cod sampled during these months consisted of coastal cod. Hence, fish belonging to the coastal cod stock may be exposed to infective *Pseudoterranova* species larvae over a longer period compared to fish belonging to the migrating ‘skrei’ stock.

Considering that ‘skrei’ arrives at the coast of Finnmark in winter after periods of extensive feeding in the open waters of the Barents Sea, the significantly lower infection level or even absence in the flesh of *Pseudoterranova* spp. in the large cod sampled in February, may indicate that the fish had ‘lost’ some of the larvae while feeding in the open sea. Indeed, we sometimes observed dead *Pseudoterranova* sp. larvae about to be disintegrated, in the flesh of several individual ‘skrei’ presently examined. We even identified molecularly a *Pseudoterranova* sp. larva from a necrotic capsule containing barely recognizable larval remains (unpublished results). Thus, a possible scenario is that the cod had eliminated the larvae by immunological means. This assumption is supported by Scott and Black (1960) who reported that encapsulated *P. decipiens* (s.l.) larvae at different stages of necrosis frequently occurred in cod more than 5 years old.

### Anisakid spatial distribution in fish hosts

The spatial distribution of *A. simplex* (s.s.) larvae followed a similar trend in all 3 gadid species examined. Larval infection levels in the flesh were similarly high in cod and saithe, carrying on average more than 13 and 18 larvae at nearly total prevalence, respectively, with roughly 21 and 34% of cod and saithe showing ⩾20 *Anisakis* larvae in the musculature. In haddock, much fewer larvae were recorded in the flesh, not exceeding 2 on average at roughly 72% prevalence.

The presently reported anisakid infection levels in the flesh of cod seem to exceed the findings of most other studies on cod from the Barents Sea and adjacent areas (Platt, 1975; Aspholm, 1995; Gay *et al*., 2018; Nadolna-Altyn *et al*., 2022). However, using the UV-press method, Bao *et al*. (2020) recorded nearly identical infection levels in the flesh of NEA migrating cod (‘skrei’) caught in March off the Lofoten archipelago in northern Norway, with almost all larvae occurring in the belly flaps. In contrast, approximately 3 times lower mean abundance of *A. simplex* (s.s) larvae were found by Gay *et al*. (2018) in the flesh of Barents Sea cod, based on UV-press on similarly sized cod as the present ones. Larval prevalence was high (>90%) and there was also a significantly positive relationship between fish size (TW) and abundance of larvae in the flesh. They also reported lower prevalence and abundance of *Pseudoterranova* spp. in the flesh of cod, compared to the pooled data including all sampling months of the present study. Moreover, Gay *et al*. (2018) also observed that *A. simplex* (s.s.) larvae tend to be slightly more abundant in the left fillet side and that the ventral fillet parts carried by far more larvae than the dorsal parts, which is consistent with the present findings and those by Bao *et al*. (2020). A similar spatial distribution of *A. simplex* (s.s.) larvae in the flesh of cod from the Norwegian Sea was recently reported by Nadolna-Altyn *et al*. (2022). Although the study was based on the candling method for nematode detection, which is considered less accurate than UV-press, *A. simplex* (s.s.) larvae appeared to occur almost exclusively in the ventral fillet parts and seemed to be significantly more abundant in the left fillet side. The larvae of *Pseudoterranova* spp. [molecularly identified as *P. decipiens* (s.s.) and *P. krabbei*] apparently showed no side preference but seemed to occur more frequently in the belly flaps. In Barents Sea haddock examined by Pierce *et al*. (2018), *A. simplex* (s.s.) larval infection level and distribution pattern in the fish flesh were largely consistent with the present findings, i.e. larval abundance was significantly higher in the ventral fillet parts (belly flaps) along with a strong lateral bias with higher numbers of larvae in the left fillet side.

The visceral organ topography of the fish host, availability of energy reserves at larval emergence in the visceral cavity (after penetration of the stomach or intestinal wall), along with the general immune-reactive state of the host, appear to be important factors determining the migration distance and final encapsulation site as well as the *in vivo* migration behaviour of the larvae (Strømnes and Andersen, 1998, 2003; Smith and Hemmingsen, 2003; Berland, 2006; Mladineo and Poljak, 2014; Strømnes, 2014). In gadids such as cod and saithe, it has been hypothesized that *A. simplex* (s.l.) encounter the liver after emerging from the stomach or intestine and become encapsulated (Berland, 2006). However, during periods of starvation the liver shrinks, leaving the stomach in direct contact with the belly flaps and, thus, further migration into the flesh may be facilitated (Smith and Hemmingsen, 2003; Berland, 2006).

Interestingly, we observed slightly lower *Anisakis* abundance in the flesh of bigger cod fished in February compared to the smaller cod in March and May, while larval abundance in the viscera of the bigger cod was more than twice as high. The slightly higher *A. simplex* (s.s.) abundance in the flesh of smaller cod in March and May even coincided with a significant decrease in HSI, i.e. the liver shrunk, with increasing season (Fig. 8). Hence, and considering the similar feeding behaviour of the fish species (Olsen *et al*., 2010), we hypothesize that since the distance between the stomach and the belly flaps is longer in bigger fish, possibly enhanced by the relatively larger liver, higher numbers of *Anisakis* may settle, and subsequently become encapsulated, in the visceral organs surrounding the stomach and foregut including the pyloric caeca and the liver (Fig. 9). The temporal variation in organ topography may similarly be responsible for the more or less pronounced bias in favour of the left side/left belly flap as primary infection site of *A. simplex* (s.s.) larvae in the flesh of the actual gadid species.

The absence of *Pseudoterranova* spp. larvae in the flesh of bigger cod in February is quite remarkable, especially considering that the samples of March and May showed 25 and 37% *Pseudoterranova* spp. prevalence, respectively. Assuming similar life histories, the immune system of the bigger cod may have eliminated the larvae that were presumably present in the flesh. In this case, the *in vivo* flesh migration of anisakid larvae in fish may primarily be an immune-evading behaviour since the relatively low fat/lipid content of the flesh of gadids does not support the hypothesis that the larvae prefer to reside in organs that are lipid-rich such as the liver.

### Food safety and quality considerations

In general, all species of *Anisakis*, *Pseudoterranova* and *Contracaecum* parasitizing marine mammals, including those presently identified, are considered zoonotic (Buchmann and Mehrdana, 2016). Additionally, allergy to *A. simplex* (s.l.) has been described in sensitized individuals following ingestion of cooked or previously frozen fish (such as cod) contaminated with dead larvae or thermostable allergens thereof (Moreno-Ancillo *et al*., 1997; Audicana and Kennedy, 2008; Audicana *et al*., 2017). Moreover, the existence of allergens and putative allergens in *P. decipiens* (s.l.) and *C. osculatum* (*s.*l.) has recently been demonstrated (Kochanowski *et al*., 2020).

In addition to the health hazardous aspect, the presence of anisakids in fish may also imply a food quality issue. In this sense, the sheer size and reddish/brownish colour of *Pseudoterranova* species larvae, if present in the edible parts of the fish such as the liver and the fillets, may be easily spotted by consumers. This parasite, vernacularly known as the ‘codworm’, has been a costly cosmetic concern for the fishing industry in Norway and Canada for many decades (Hemmingsen *et al*., 1993). The larvae of *Anisakis* species, on the other hand, are not that easily spotted when situated in the fillets due to their nearly transparent appearance, unless the surrounding capsule tissue is melanized. Based on this awareness, the fish processing industry is obliged by EU regulations to inspect fishery products for the presence of visible parasites, and no obviously parasitized product must be put on the market (EC, 2004). In this respect, the present results on the distribution pattern of *A. simplex* (s.s.) larvae in the flesh of the actual gadid species showing highest larval abundance in the belly flaps could be useful information for the fish processing industry. Hence, in order to minimize the risk of anisakid larvae to be present in the products of NEA cod, saithe and haddock, trimming of the flesh by removing the belly flaps could reduce larval occurrence in the fillets by up to 86% in saithe, and even ⩾90% in cod and haddock.

## Concluding remarks

The presently reported apparently higher abundances of larval anisakids, especially for *A. simplex* (s.s.) in cod, compared to other recent studies, may suggest that the biomass of actual anisakids in the southern Barents Sea is about to increase, particularly with regards to *A. simplex* (s.s.). For example, some evidence exists that the distribution and migration routes of certain cetaceans have changed in some areas of the NE Atlantic (Simmonds and Eliott, 2009). Indeed, several species of ‘oceanic’ dolphins (Delphinidae), acting as definitive hosts for *A. simplex* (s.s.), have recently changed their distribution range by following their prey into more northerly waters including the Barents Sea (Evans and Bjørge, 2013). Thus, a possible scenario is that increased numbers of cetaceans may stay relatively longer in Arctic or Subarctic waters due to more favourable conditions including higher average sea temperatures during the warmer months. This again may facilitate higher larval output into the ecosystem, along with extended favourable conditions for hatching and host finding of infective anisakid larvae.

Thus, anisakid nematodes are useful indicators that may contribute to detect long- and short-term changes in the ecosystem since their life cycle involves organisms at low (plankton or semi-plankton), intermediate (fish and squid) and top level (marine mammals) of the food web (Mattiucci *et al*., 2017). Surveillance of these parasites on a regular basis, especially in commercially harvested fish from the Barents Sea, could therefore provide valuable information, not only concerning the possible impact increased larval occurrence could have on food safety and product quality, but also for assessing the general state of the ecosystem in terms of intact and functioning trophic interrelationships in time and space.

## References

[ref1] Aspholm PE (1995) *Anisakis simplex* Rudolphi, 1809, infection in fillets of Barents Sea cod *Gadus morhua* L. Fisheries Research 23, 375–379.

[ref2] Audicana MT and Kennedy MW (2008) *Anisakis simplex*: from obscure infectious worm to inducer of immune hypersensitivity. Clinical Microbiology Reviews 21, 360–379. 18400801 10.1128/CMR.00012-07 PMC2292572

[ref3] Audicana M, Girao I and Longo N (2017) *Anisakis simplex*, a new hero in the anaphylaxis scene. SM Emergency Medicine and Critical Care 1, 1008.

[ref4] Bao M, Pierce GJ, Strachan NJC, Pascual S, González-Muñoz M and Levsen A (2019) Human health, legislative and socioeconomic issues caused by the fish-borne zoonotic parasite *Anisakis*: challenges in risk assessment. Trends in Food Science & Technology 86, 298–310.

[ref5] Bao M, Cipriani P, Giulietti L, Sunde Roiha I, Paoletti M, Palomba M and Levsen A (2020) Air-dried stockfish of Northeast Arctic cod do not carry viable anisakid nematodes. Food Control 116, 107322.

[ref6] Bao M, Cipriani P, Giulietti L, Drivenes N and Levsen A (2021) Quality issues related to the presence of the fish parasitic nematode *Hysterothylacium aduncum* in export shipments of fresh Northeast Arctic cod (*Gadus morhua*). Food Control 121, 107724.

[ref7] Berland B (1961) Nematodes from some Norwegian marine fishes. Sarsia 2, 1–50.

[ref8] Berland B (2006) Musing on nematode parasites. Fisken og Havet 11, 1–26.

[ref9] Berland B (1989) Identification of fish larval nematodes from fish. In Möller H (ed). Nematode problems in north Atlantic fish. Report from a workshop in Kiel 3–4 April 1989, pp. 16–22.

[ref10] Bogstad B, Gjøsæter H, Haug T and Lindstrøm U (2015) A review of the battle for food in the Barents Sea: cod vs marine mammals. Frontiers in Ecology and Evolution 3, 29.

[ref11] Buchmann K and Mehrdana F (2016) Effects of anisakid nematodes *Anisakis simplex* (s. l.), *Pseudoterranova decipiens* (s.l.) and *Contracaecum osculatum* (s.l.) on fish and consumer health. Food and Waterborne Parasitology 4, 13–22.

[ref12] Bush AO, Lafferty KD, Lotz JM and Shostak AW (1997) Parasitology meets ecology on its own terms: Margolis et al. revisited. Journal of Parasitology 83, 575. 9267395

[ref13] Cipriani P, Palomba M, Giulietti L, Marcer F, Mazzariol S, Santoro M, Alburqueque RA, Covelo P, López A, Santos MB, Pierce GJ, Brownlow A, Davison NJ, McGovern B, Frantzis A, Alexiadou P, Højgaard DP, Mikkelsen B, Paoletti M, Nascetti G, Levsen A and Mattiucci S (2022) Distribution and genetic diversity of *Anisakis* spp. in cetaceans from the Northeast Atlantic Ocean and the Mediterranean Sea. Scientific Reports 12, 13664. 35953527 10.1038/s41598-022-17710-1 PMC9372146

[ref14] Directorate of Fisheries (2022) Economic and biological figures from Norwegian fisheries – 2021. ISSN/ISSB: 2464-3157. Retrieved from Directorate of Fisheries website https://www.fiskeridir.no/Yrkesfiske/Tall-og-analyse/Statistiske-publikasjoner/Noekkeltall-for-de-norske-fiskeriene (Accessed 30 May 2022).

[ref15] EC (2004) Regulation (EC) No 853/2004 of the European Parliament and of the Council of 29 April 2004 laying down specific hygiene rules for food of animal origin. Official Journal of the European Union 30.4.2004, 151.

[ref16] Evans PGH and Bjørge A (2013) Impacts of climate change on marine mammals. MCCIP Science Review 2013, 134–148.

[ref17] Gay M, Bao M, MacKenzie K, Pascual S, Buchmann K, Bourgau O, Couvreur C, Mattiucci S, Paoletti M, Hastie LC, Levsen A and Pierce GJ (2018) Infection levels and species diversity of ascaridoid nematodes in Atlantic cod, *Gadus morhua*, are correlated with geographic area and fish size. Fisheries Research 202, 90–102.

[ref18] Hemmingsen W, Lysne D, Eidnes T and Skorping A (1993) The occurrence of larval ascaridoid nematodes in wild-caught and in caged and artificially fed Atlantic cod, *Gadus morhua* L., in Norwegian waters. Fisheries Research 15, 379–386.

[ref19] Hemmingsen W, Halvorsen O and MacKenzie K (2000) The occurrence of some metazoan parasites of Atlantic cod, *Gadus morhua* L., in relation to age and sex of the host in Balsfjord (70°N), North Norway. Polar Biology 23, 368–372.

[ref20] ICES (2011) Report of the Working Group on Harp and Hooded Seals (WGHARP), 15–19 August 2011, St. Andrews, Scotland, UK. ICES CM 2011/ACOM:22. pp. 78.

[ref21] Kahl W (1939) Nematoden in Seefischen III. Statistische Erhebungen über den Nematodenbefall von Seefischen. Zeitschrift für Parasitenkunde 11, 16–41.

[ref22] Karpiej K, Dzido J, Rokicki J and Kijewska A (2013) Anisakid nematodes of Greenland halibut *Reinhardtius hippoglossoides* from the Barents Sea. Journal of Parasitology 99, 650. 23536987 10.1645/GE-2987.1

[ref23] Kjesbu OS, Bogstad B, Devine JA, Gjøsæter H, Howell D, Ingvaldsen RB, Nash RDM and Skjæraasen JE (2014) Synergies between climate and management for Atlantic cod fisheries at high latitudes. Proceedings of the National Academy of Sciences 111, 3478–3483. 10.1073/pnas.1316342111 PMC3948268 24550465

[ref24] Kochanowski M, Dąbrowska J, Różycki M, Karamon J, Sroka J and Cencek T (2020) Proteomic profiling reveals new insights into the allergomes of *Anisakis simplex*, *Pseudoterranova decipiens*, and *Contracaecum osculatum*. Journal of Parasitology 106, 572. 32906150 10.1645/19-75

[ref25] Levsen A, Paoletti M, Cipriani P, Nascetti G and Mattiucci S (2016) Species composition and infection dynamics of ascaridoid nematodes in Barents Sea capelin (*Mallotus villosus*) reflecting trophic position of fish host. Parasitology Research 115, 4281–4291. 27473836 10.1007/s00436-016-5209-9

[ref26] Levsen A, Svanevik CS, Cipriani P, Mattiucci S, Gay M, Hastie LC, Bušelić I, Mladineo I, Karl H, Ostermeyer U, Buchmann K, Højgaard D, González AF, Pascual S and Pierce GJ (2018) A survey of zoonotic nematodes of commercial key fish species from major European fishing grounds – introducing the FP7 PARASITE exposure assessment study. Fisheries Research 202, 4–21.

[ref27] Mattiucci S and Nascetti G (2008) Advances and trends in the molecular systematics of anisakid nematodes, with implications for their evolutionary ecology and host-parasite coevolutionary processes. Advances in Parasitology 66, 47–148. 18486689 10.1016/S0065-308X(08)00202-9

[ref28] Mattiucci S, Cipriani P, Webb SC, Paoletti M, Marcer F, Bellisario B, Gibson DI and Nascetti G (2014) Genetic and morphological approaches distinguish the three sibling species of the *Anisakis simplex* species complex, with a species designation as *Anisakis berlandi* n. sp. for *A. simplex* sp. C (Nematoda: Anisakidae). Journal of Parasitology 100, 199–214. 24224764 10.1645/12-120.1

[ref29] Mattiucci S, Cipriani P, Paoletti M, Levsen A and Nascetti G (2017) Reviewing biodiversity and epidemiological aspects of anisakid nematodes from the North-east Atlantic Ocean. Journal of Helminthology 91, 422–439. 28397641 10.1017/S0022149X1700027X

[ref30] Mattiucci S, Cipriani P, Levsen A, Paoletti M and Nascetti G (2018) Molecular epidemiology of *Anisakis* and Anisakiasis: an ecological and evolutionary road map. Advances in Parasitology 99, 93–263. 29530312 10.1016/bs.apar.2017.12.001

[ref31] Mercken E, Van Damme I, Serradell A and Gabriël S (2020) Presence of Anisakidae in commercial fish species imported into the Belgian food markets: a systematic review and meta-analyses. International Journal of Food Microbiology 318, 108456. 31821936 10.1016/j.ijfoodmicro.2019.108456

[ref32] Mercken E, Van Damme I, Šoba B, Vangeenberghe S, Serradell A, Lumain JPL, De Sterck T, Lalle M and Gabriël S (2021) High occurrence of Anisakidae at retail level in cod (*Gadus morhua*) belly flaps and the impact of extensive candling. Food and Waterborne Parasitology 22, e00108. 33681486 10.1016/j.fawpar.2020.e00108 PMC7930124

[ref33] Mishin TV (2021) Cetaceans of the Barents Sea: fauna and population status at the beginning of the XXI century. Marine Biological Journal 6, 52–68.

[ref34] Mladineo I and Poljak V (2014) Ecology and genetic structure of zoonotic *Anisakis* spp. from Adriatic commercial fish species. Applied and Environmental Microbiology 80, 1281–1290. 24317085 10.1128/AEM.03561-13 PMC3911056

[ref35] Moreno-Ancillo A, Caballero MT, Cabañas R, Contreras J, Martin-Barroso JA, Barranco P and López-Serrano MC (1997) Allergic reactions to *Anisakis simplex* parasitizing seafood. Annals of Allergy, Asthma & Immunology 79, 246–250. 10.1016/S1081-1206(10)63009-8 9305232

[ref36] Münster J, Klimpel S, Fock HO, MacKenzie K and Kuhn T (2015) Parasites as biological tags to track an ontogenetic shift in the feeding behaviour of *Gadus morhua* off West and East Greenland. Parasitology Research 114, 2723–2733. 10.1007/s00436-015-4479-y. 25899328

[ref37] Nadler SA and Hudspeth DSS (2000) Phylogeny of the Ascaridoidea (Nematoda: Ascaridida) based on three genes and morphology hypotheses of structural and sequence evolution. Journal of Parasitology 86, 380–393. 10780561 10.1645/0022-3395(2000)086[0380:POTANA]2.0.CO;2

[ref38] Nadolna-Ałtyn K, Podolska M, Pawlak J and Szostakowska B (2022) Distribution of anisakid nematodes in the muscle tissue of cod (*Gadus morhua*) from the Norwegian Sea. Oceanologia 64, 489–502. 10.1016/j.oceano.2022.03.003

[ref39] Najda K, Kijewska A, Kijewski T, Plauška K and Rokicki J (2018) Distribution of ascaridoid nematodes (Nematoda: Chromadorea: Ascaridoidea) in fish from the Barents Sea. Oceanological and Hydrobiological Studies 47, 128–139.

[ref40] Nascetti G, Cianchi R, Mattiucci S, D'amelio S, Orecchia P, Paggi L, Brattey J, Berland B, Smith JW and Bullini L (1993) Three sibling species within *Contracaecum osculatum* (Nematoda, Ascaridida, Ascaridoidea) from the Atlantic Arctic-boreal region: reproductive isolation and host preferences. International Journal for Parasitology 23, 105–120. 8468125 10.1016/0020-7519(93)90103-6

[ref41] Nordøy ES, Folkow LP, Potelov V, Prischemikhin V and Blix AS (2008) Seasonal distribution and dive behaviour of harp seals (*Pagophilus groenlandicus*) of the White Sea–Barents Sea stock. Polar Biology 31, 1119–1135.

[ref42] Olsen E, Aanes S, Mehl S, Holst JC, Aglen A and Gjøsæter H (2010) Cod, haddock, saithe, herring, and capelin in the Barents Sea and adjacent waters: a review of the biological value of the area. ICES Journal of Marine Science 67, 87–101.

[ref43] Pierce GJ, Bao M, MacKenzie K, Dunser A, Giulietti L, Cipriani P, Mattiucci S and Hastie LC (2018) Ascaridoid nematode infection in haddock (*Melanogrammus aeglefinus*) and whiting (*Merlangius merlangus*) in Northeast Atlantic waters. Fisheries Research 202, 122–133.

[ref44] Platt NE (1975) Infestation of cod (*Gadus morhua* L.) with larvae of codworm (*Terranova decipiens* Krabbe) and herringworm, *Anisakis* sp. (Nematoda Ascaridata), in north Atlantic and Arctic waters. Journal of Applied Ecology 12, 437.

[ref45] Scott DM and Black WF (1960) Studies on the life-history of the ascarid *Porrocaecum decipiens* in the Bras d'Or Lakes, Nova Scotia, Canada. Journal of the Fisheries Research Board of Canada 17, 763–774.

[ref46] Simmonds MP and Eliott WJ (2009) Climate change and cetaceans: concerns and recent developments. Journal of the Marine Biological Association of the United Kingdom 89, 203–210. 10.1017/S0025315408003196.

[ref47] Skarstein TH, Westgaard JI and Fevolden SE (2007) Comparing microsatellite variation in north-east Atlantic cod (*Gadus morhua* L.) to genetic structuring as revealed by the pantophysin (*Pan* I) locus. Journal of Fish Biology 70, 271–290.

[ref48] Smith JW and Hemmingsen W (2003) Atlantic cod *Gadus morhua* L.: visceral organ topography and the asymmetrical distribution of larval ascaridoid nematodes in the musculature. Ophelia 57, 137–144.

[ref49] Sobecka E, Luczak E, Wiecaszek B and Antoczek A (2011) Parasite community structure of cod from Bear Island (Barents Sea) and Pomeranian Bay (Baltic Sea). Polish Polar Research 32, 253–262.

[ref50] Strømnes E (2014) An *in vitro* study of lipid preference in whaleworm (*Anisakis simplex*, Nematoda, Ascaridoidea, Anisakidae) third-stage larvae. Parasitology Research 113, 1113–1118. 24458651 10.1007/s00436-013-3748-x PMC3932165

[ref51] Strømnes E and Andersen K (1998) Distribution of whaleworm (*Anisakis simplex*, Nematoda, Ascaridoidea) L3 larvae in three species of marine fish; saithe (*Pollachius virens* (L.)), cod (*Gadus morhua* L.) and redfish (*Sebastes marinus* (L.)) from Norwegian waters. Parasitology Research 84, 281–285. 9569092 10.1007/s004360050396

[ref52] Strømnes E and Andersen K (2003) Growth of whaleworm (*Anisakis simplex*, Nematodes, Ascaridoidea, Anisakidae) third-stage larvae in paratenic fish hosts. Parasitology research 89, 335–341. 12632142 10.1007/s00436-002-0756-7

